# Elevated Levels of Pentraxin 3 Correlate With Neutrophilia and Coronary Artery Dilation During Acute Kawasaki Disease

**DOI:** 10.3389/fped.2020.00295

**Published:** 2020-06-25

**Authors:** Lauren L. Ching, Vivek R. Nerurkar, Eunjung Lim, Ralph V. Shohet, Marian E. Melish, Andras Bratincsak

**Affiliations:** ^1^Department of Tropical Medicine, Medical Microbiology and Pharmacology, John A. Burns School of Medicine, University of Hawai'i at Mānoa, Honolulu, HI, United States; ^2^Pacific Center for Emerging Infectious Diseases Research, John A. Burns School of Medicine, University of Hawai'i at Mānoa, Honolulu, HI, United States; ^3^Biostatistics Core Facility, Department of Quantitative Health Sciences, John A. Burns School of Medicine, University of Hawai'i at Mānoa, Honolulu, HI, United States; ^4^Department of Medicine, John A. Burns School of Medicine, University of Hawai'i at Mānoa, Honolulu, HI, United States; ^5^Department of Pediatrics, John A. Burns School of Medicine, University of Hawai'i at Mānoa, Honolulu, HI, United States; ^6^Kapi‘olani Medical Specialists, Hawai'i Pacific Health, Honolulu, HI, United States

**Keywords:** Kawasaki disease, pentraxin 3, coronary artery lesions, coronary artery dilatation, coronary artery aneurysm, IVIG resistance

## Abstract

Kawasaki disease (KD) is the leading cause of acquired pediatric heart disease in the developed world as 25–30% of untreated patients and at least 5% of treated patients will develop irreversible coronary artery lesions (CAL). Pentraxin-3 (PTX-3) has been well-studied in inflammatory diseases, particularly in cardiovascular diseases associated with vascular endothelial dysfunction. We hypothesized that PTX-3 plays an important role in the development of KD-associated CAL and investigated the circulating levels of PTX-3 in the serum of KD patients. Children with acute KD were followed from diagnosis through normalization of the clinical parameters of inflammation (convalescent phase). Serum samples were obtained and echocardiograms were conducted at several phases of the illness: acute [prior to intravenous immunoglobulin (IVIG) treatment], sub-acute (5–10 days after IVIG treatment), and convalescent (1–4 months after KD diagnosis). Seventy children were included in the final cohort of the study, of whom 26 (37%) presented with CAL and 18 (26%) developed IVIG resistance. The patients included in this study came from diverse ethnic backgrounds, mostly with mixed ancestry/ ethnicity. Significantly increased PTX-3 levels were observed during the acute phase of KD compared to the sub-acute and the convalescent phases. The PTX-3 levels during acute KD were significantly higher among KD patients with CAL compared to patients with normal coronary arteries (NCA). Also, the PTX-3 levels were significantly higher in patients with IVIG resistance. Furthermore, the PTX-3 levels were significantly higher in IVIG-resistant KD patients with CAL as compared to the NCA group. Moreover, the PTX-3 levels were significantly correlated to coronary artery z-score during acute KD and to neutrophil counts throughout KD progression regardless of coronary artery z-score. Elevated PTX-3 levels correlated to elevated neutrophil counts, a known source of PTX-3 in acute inflammation and an important player in the development of KD vasculitis. We, therefore, suggest PTX-3 as a novel factor in the development of KD-associated CAL and propose neutrophil-derived PTX-3 as contributing to KD vascular dysfunction.

## Introduction

Kawasaki disease (KD) is the leading cause of acquired pediatric heart disease in the developed world, presenting in young children as acute, febrile, self-limiting, systemic vasculitis (1, 2). The clinical and epidemiological features of KD have suggested an infectious cause (3). However, the etiology of the disease remains unknown. Clinically, KD manifests with prolonged high fever, a typical rash, conjunctivitis, and lymphadenopathy (4). The vasculitis in KD particularly affects the coronary arteries. If not recognized and treated within the first 7–10 days of illness, there is a 25% chance of lasting damage to the coronary arteries, often with aneurysm formation, which can lead to death due to coronary artery thrombosis or rupture (5). The case fatality rate for KD in Japan is <0.01% (6). Even with prompt recognition and recommended treatment—intravenous immunoglobulin (IVIG) and high-dose aspirin−30% of KD children develop transient coronary artery lesions (CAL), and about 5% have persistent CAL or aneurysms after recovery from the illness. Persistent coronary damage from KD can cause serious complications, including death (7–10).

A specific diagnostic test for timely and accurate identification of KD would be a boon to clinicians, facilitating early treatment and reduction of the risk for development of coronary involvement. Previous studies have demonstrated the risk for CAL in KD patients who present with higher baseline coronary artery dimensions, exhibit IVIG resistance (persistent fever for more than 24 h after initial IVIG infusion), or have a delay in diagnosis causing a delay in treatment (11–15). Several algorithms have been developed in Japan to predict IVIG resistance and subsequent coronary damage; these include the Kobayashi (16), Egami (17), Sano (18), and Harada (19) scoring systems. These incorporate demographic, clinical, and laboratory parameters. While these scoring systems are useful in the Japanese population, their efficacy is lost in populations outside of Japan (4, 20). As a result, clinicians in other parts of the world do not uniformly use these Japanese scoring systems. Son and colleagues described the great need for improved methods/scoring systems to identify KD patients at risk for coronary aneurysms, demonstrating the high predictive value of a maximum z-score of ≥2.0 (20).

Current theories of KD pathogenesis include (i) infection with classic immune responses to an as-yet identified pathogen(s), (ii) an autoantibody or T-cell driven autoimmune response triggered by an antigen, i.e., molecular mimicry, and (iii) an autoinflammatory response, which includes innate immune responses that cause systemic inflammation as well as damage to the coronary arterial wall (3). Previous studies have focused on cytokines and chemokines during acute KD. However, these proteins are relatively non-specific and are elevated in many inflammatory processes (21, 22).

PTX-3 is a member of the pentraxin protein family, a class of soluble pattern recognition receptors (PRR), which includes C-reactive protein (CRP), a widely used laboratory parameter of clinical inflammation, and serum amyloid P component (23). This family of proteins plays integral roles in complement activation, amplification, and regulation (24). PTX-3 is thought to have a role in several processes in the cardiovascular system, including inflammation, angiogenesis, tumorigenesis, and cell adhesion (25). PTX-3 is a soluble PRR associated with the local activation of the innate immune system and inflammation. Studies of adult coronary artery disease have identified PTX-3 as a predictor of all-cause mortality, cardiac death, and cardiac events (26). High levels of immune and endothelial cell-derived PTX-3 have been associated with coronary artery dysfunction and adverse outcomes, with correlations to CRP and MMP9 levels (27). PTX-3 is also associated with vascular endothelial dysfunction and morphological alterations through impairment of the nitric oxide pathway (28). From these studies, we hypothesized that PTX-3 might play a role in KD pathogenesis and could be employed as a diagnostic or prognostic marker of KD. We investigated the circulating PTX-3 levels in the serum of KD patients with and without CAL. Here we report elevated levels of PTX-3 during the acute phase of KD, particularly in association with CAL. These data suggest a role for PTX-3 in KD pathogenesis, specifically the vascular damage that leads to CAL in the most severe cases of KD.

## Materials and Methods

### Study Population and Laboratory Evaluation

All children recruited in this study were admitted to the KMCWC in Honolulu, Hawai‘i, between October 2013 and November 2018. The children were evaluated by experienced clinicians at KMCWC and fulfilled the diagnostic criteria for KD as endorsed by the American Heart Association (AHA). All patients were treated, per AHA guidelines, with IVIG and high-dose acetylsalicylic acid as primary treatment. IVIG resistance was defined as patients having a persistent or recurrent fever for more than 36 h after completion of the initial IVIG treatment. Serum was separated within 24 h of blood collection and stored at −80°C until further analysis. The serum samples for research were collected from a total of 70 patients at three phases: 62 patients at the time of KD diagnosis/disease onset and prior to IVIG treatment (acute phase), 65 patients at 1 to 2 weeks following IVIG treatment (sub-acute phase), and 64 patients at 3 weeks to 4 months following disease onset (convalescent phase). There were 53 patients whose samples were obtained at all three phases. Convalescence was defined by the normalization of the clinical laboratory features of KD such as elevated erythrocyte sedimentation rate (ESR), C-reactive protein (CRP) levels, and white blood cell (WBC) counts. In addition to these parameters, echocardiogram measurements and additional clinical laboratory data [i.e., ESR, CRP, complete blood count with differential hemoglobin (Hgb), hematocrit (Hct), red blood cell (RBC) count, WBC count, absolute lymphocyte count (ALC), absolute neutrophil count (ANC), absolute eosinophil count, absolute basophil count, absolute monocyte count (MO), and platelet (PLT) count] were evaluated at each phase of the disease. The echocardiogram measurements were collected for all patients at each phase of the disease. However, not all clinical laboratory parameters were captured at each phase of the disease in all patients.

### Echocardiogram Measurements

A complete echocardiogram was conducted as part of the standard diagnostic evaluation of KD for all patients at each phase of the disease. The inner diameter of the right coronary artery (RCA) and the left anterior descending coronary artery (LAD) was measured in the modified parasternal views, and the z-score of the coronary artery diameters was calculated based on the patient's body surface area using the Boston dataset for standards (29). CAL was defined as having either an RCA or LAD z-score ≥2.5 at any phase of KD (30).

### Protein Expression Analysis

Circulating levels of PTX-3 were measured in the patient's serum samples by MILLIPLEX MAP Human Cardiovascular Disease Multiplex Assay (Cat. No. HCVD4MAG-67K; MilliporeSigma, Burlington, MA, USA) following the manufacturer's instructions. The patient's samples were tested in duplicate. Plates were analyzed on a Luminex 200 system (Luminex Corp., Austin, TX, USA) and data were analyzed using xPONENT 3.1 (Luminex Corp.) and Milliplex Analyst 5.1 Software (MilliporeSigma).

### Statistical Analysis

The patient's clinical and demographic characteristics were summarized by descriptive analysis such as median and interquartile range (IQR) for continuous variables and frequency and percentage for categorical variables. Repeated-measures analysis of variance (ANOVA) was used to characterize how clinical laboratory parameters and PTX-3 levels change over the course of KD pathogenesis (i.e., acute, sub-acute, and convalescence) (within-factor) and CAL (between-factor), accounting for within-subject dependence. Model diagnostics were explored (e.g., *Q*–*Q* plot and residual plots) and the outcome variables were transformed by natural logarithm to satisfy the model assumption (normality and constant variance). With the final model, Tukey's *post-hoc* test was conducted to compare the different phases. Spearman's correlation was computed to assess bivariate associations between PTX-3 and coronary artery z-score or clinical laboratory variables at each phase of KD. Fisher's exact test was used to evaluate the IVIG response group effect and computed odds ratio. Repeated-measures ANOVA, Spearman's correlations, and Fisher's exact test were conducted using GraphPad Prism version 8.4.0 (GraphPad Software, San Diego, CA, USA). To assess the association between immune factors and laboratory variables over the clinical course of KD, repeated-measures correlations were calculated using the package (31) in R version 3.5.3. Repeated-measures correlation assesses the association between paired variables measured multiple times within subjects. A heat map was generated to depict repeated-measures and Spearman's correlation. The number in each cell in the heat map indicates the repeated-measures correlation between the column and the row variables. The cells in shades of red show positive correlations and the cells in shades of blue show negative correlations.

## Results

### Clinicoepidemiological Characteristics of KD Patients

A total of 222 patients were eligible and 70 patients were enrolled in this study. The patient population consists of an almost equal number of boys (*n* = 34, 49%) and girls (*n* =36, 51%), with 86% (*n* =60) under 5 years of age (Table 1). The study population includes a variety of self-reported whole or partial ethnic backgrounds, with the majority of patients from Asian ancestry (*n* =58, 83%), and half of the patients reported two or more ethnicities (up to five) (*n* =35, 50%) (Table 1). Most patients (*n* = 68, 97%) received primary treatment within the first 10 days of onset of fever; IVIG resistance was observed in 18 patients (26%) (Table 1).

**Table 1:** Patients' clinicoepidemiological characteristics.

**Variables**	**All (*****n*** **=** **70)**		**Normal coronary arteries (*****n*** **=** **44)**		**Coronary artery lesions (*****n*** **=** **26)**	
**Gender**						
Male	*n* = 34	(49%)	*n* = 18	(41%)	*n* = 16	(62%)
Female	*n* = 36	(51%)	*n* = 26	(59%)	*n* = 10	(38%)
Age in years, median (IQR)	2.63 (1.00–3.90)		2.25 (0.83–3.46)		3.21 (1.33–4.63)	
<1	*n* = 17	(24%)	*n* = 12	(27%)	*n* = 5	(19%)
1–5	*n* = 43	(61%)	*n* = 28	(64%)	*n* = 15	(58%)
>5	*n* = 10	(14%)	*n* = 4	(9%)	*n* = 6	(23%)
**Ethnicity**^**a**^						
Asian	*n* = 58	(83%)	*n* = 37	(84%)	*n* = 21	(81%)
NHOPI	*n* = 30	(43%)	*n* = 19	(43%)	*n* = 11	(42%)
White	*n* = 29	(41%)	*n* = 22	(50%)	*n* = 7	(27%)
Hispanic or Latino	*n* = 5	(7%)	*n* = 3	(7%)	*n* = 2	(8%)
AI/AN	*n* = 2	(3%)	*n* = 1	(2%)	*n* = 1	(4%)
Black or African American	*n* = 2	(3%)	*n* = 2	(5%)	*n* = 0	(0%)
Other	*n* = 1	(1%)	*n* = 1	(2%)	*n* = 0	(0%)
Two or more ethnicities	*n* = 35	(50%)	*n* = 22	(50%)	*n* = 13	(50%)
**Clinical features**						
Duration of fever in days, median (IQR)	5.00 (4.00–7.00)		5.00 (4.00–6.00)		6.00 (5.00–8.00)	
Interval between fever onset and treatment administration in days, median (IQR)	5.00 (4.00–6.00)		4.00 (3.00–6.00)		5.00 (4.00–8.00)	
IVIG treatment within 10 days of fever onset	*n* = 68	(97%)	*n* = 43	(98%)	*n* = 25	(96%)
IVIG resistance	*n* = 18	(26%)	*n* = 5	(11%)	*n* = 13	(50%)
CAL z-score ≥2.50^b^	*n* = 26	(37%)	*n* = 0	(0%)	*n* = 26	(100%)
CAL z-score^b^, median (IQR)	4.23 (3.37–4.7)		1.48 (1.12–1.87)		4.16 (3.35–4.70)	

SEM, standard error mean; IQR, interquartile range; AI/AN, American Indian/American native; NHOPI, Native Hawaiian or Pacific Islander; Other, other not specified.

a Can have multiple ethnicities.

b *Number of children whose z-score of the right coronary artery or the left anterior descending artery is equal or >2.5 at either acute or sub-acute visits*.

This study included 26 (37%) patients who presented with CAL (Table 1). Among patients with CAL, maximal coronary artery z-scores occurred most frequently during the acute phase [*n* = 15; 3.96 (3.27–4.35)], with fewer during the sub-acute phase [*n* = 11; 4.38 (3.81–7.40)], and none at the convalescent phase. Clinicoepidemiological characteristics were reported among patients with normal coronary arteries (NCA) and CAL. We did not observe any significant differences in clinicoepidemiological characteristics among the NCA and the CAL groups, with the exception of IVIG resistance placing children at a higher risk for CAL (OR = 7.8; 95% CI 2.38–23.54, *p* < 0.001). However, in our study population, males accounted for more of the CAL patients (*n* = 16, 62%) as compared to females (*n* = 10, 38%), and White children were less susceptible to CAL (*n* = 7, 27%) as compared to non-White children (*n* = 19; 73%).

### Elevated Levels of PTX-3 During Acute KD

**Figure 1:**
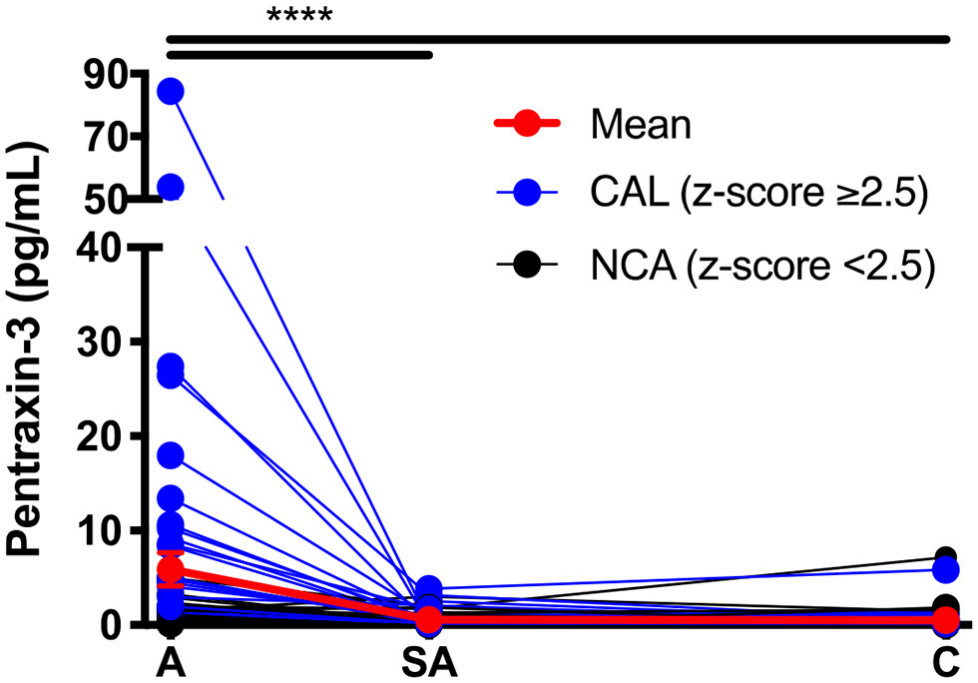
Circulating levels of PTX-3 throughout Kawasaki disease (KD) progression. Repeated-measures ANOVA was used to compare the PTX-3 levels between the three phases of KD. The black lines track the KD patients with normal coronary artery (*n* = 44), the blue lines track the KD patients with coronary artery lesion (*n* = 26), and the red line tracks the mean ± SEM of all the patient serum samples (*n* = 70) evaluated at each phase of the disease. A, acute (*n* = 62); SA, sub-acute (*n* = 65); C, convalescent (*n* = 64); SEM, standard error mean. ***p ≤ 0.001.

The KD patients were evaluated for circulating levels of PTX-3 throughout the clinical course of the disease. This analysis revealed a significant increase in PTX-3 levels in the acute (5.87 ± 1.76 pg/ml, 0.08–84.53) as compared to the sub-acute (0.58 ± 0.10 pg/ml, 0.05–3.84) and the convalescent (0.54 ± 0.82 pg/ml, 0.04–7.18) KD phases (Figure 1, Table 2). The CRP levels and the WBC and the ANC counts peaked in the acute phase and returned to normal levels in the convalescent phase. The ESR levels were elevated in the acute phase, peaked in the sub-acute phase, and returned to normal in the convalescent phase. Furthermore, red blood cell parameters such as RBC, Hgb, and Hct and MO count significantly decreased in the acute phase and peaked in the convalescent phase. The ALC and PLT counts peaked in the sub-acute phase. The lymphocyte count remained elevated in the convalescent phase (Table 2, Supplementary Figure 1).

**Table 2:** PTX-3, coronary artery z-scores, and clinical assessment of inflammation throughout Kawasaki disease (KD) progression.

	**Acute (*****n*** **=** **70)**		***n***	**Sub-acute (*****n*** **=** **69)**		***n***	***P*^**a**^**	**Convalescent (*****n*** **=** **66)**		***n***	***P*^**b**^**
PTX-3 (pg/ml)	5.87 ± 1.76	(0.08–84.53)	62	0.58 ± 0.10	(0.05–3.84)	65	^***^	0.54 ± 0.82	(0.04–7.18)	64	^***^
Right coronary artery z-score	1.63 ± 0.38	(−2.35–20.83)	70	1.55 ± 0.43	(−2.40–24.03)	69	ns	1.02 ± 0.47	(−1.92–2.25)	66	^**^
Left anterior descending coronary artery z-score	2.40 ± 0.50	(−3.00–26.24)	70	2.20 ± 0.69	(−3.00–36.30)	69	^*^	1.62 ± 0.74	(−2.25–37.23)	66	^***^
CA z-score MAX	2.75 ± 0.49	(−2.35–26.24)	70	2.72 ± 0.68	(−2.34–36.30)	69	ns	2.03 ± 0.72	(−1.00–37.23)	66	^***^
Erythrocyte sedimentation rate (mm/h)	78.38 ± 3.40	(28.02–120.0)	68	85.32 ± 3.85	(6.00–120.0)	66	ns	14.39 ± 1.69	(1.00–84.00)	66	^***^
C-reactive protein (mg/L)	110 ± 10.16	(84.39–427.0)	69	7.45 ± 1.23	(0.00–42.60)	66	^***^	1.14 ± 0.28	(0.00–11.40)	60	^***^
Hemoglobin (g/dl)	10.97 ± 0.13	(1.11–13.50)	70	10.71 ± 0.13	(8.30–13.30)	67	ns	12.16 ± 0.12	(8.90–14.80)	66	^***^
Hematocrit (g/dl)	32.60 ± 0.36	(3.00–38.40)	69	32.16 ± 0.37	(25.00–38.50)	67	ns	36.21 ± 0.32	(29.20–43.20)	66	^***^
Red blood cell (10^12^/L)	4.09 ± 0.05	(0.41–4.99)	69	3.99 ± 0.05	(2.89–4.90)	66	ns	4.55 ± 0.05	(3.69–5.35)	66	^***^
White blood cell (10^9^/L)	14.97 ± 0.75	(6.24–42.00)	70	11.66 ± 0.52	(4.20–25.60)	67	^***^	9.00 ± 0.31	(3.50–17.50)	66	^***^
Absolute lymphocyte count (10^9^/L)	3.11 ± 0.23	(1.86–8.40)	63	5.70 ± 0.27	(1.89–11.10)	65	^***^	5.19 ± 0.27	(1.41–11.55)	64	^***^
Absolute neutrophil count (10^9^/L)	11.39 ± 1.10	(8.83–63.20)	64	4.90 ± 0.47	(0.67–17.66)	63	^***^	3.01 ± 0.21	(0.74–8.78)	65	^***^
Absolute eosinophil count (10^9^/L)	0.40 ± 0.05	(0.35–1.38)	49	0.44 ± 0.05	(0.01–1.66)	57	ns	0.38 ± 0.05	(0.03–2.28)	61	ns
Absolute basophil count (10^9^/L)	0.07 ± 0.01	(0.08–0.41)	29	0.11 ± 0.02	(0.02–0.37)	22	ns	0.08 ± 0.01	(0.02–0.20)	40	ns
Monocyte (10^9^/L)	0.76 ± 0.07	(0.54–2.80)	61	0.75 ± 0.06	(0.10–2.02)	63	ns	2.39 ± 0.39	(0.07–14.00)	65	^**^
Platelet (10^9^/L)	383 ± 15.79	(132.1–699.0)	70	633 ± 22.57	(302.0–1,365)	67	^***^	380 ± 13.9	(207.0–743.0)	66	ns

Mean ± SEM (range) evaluated for the indicated variable of all the patient serum samples (n = 70) at the acute, sub-acute, and convalescent phases of the disease. Repeated-measures ANOVA was used to compare the coronary artery z-scores and the clinical laboratory parameters between the three phases of KD. p-value comparing the effect of the variable at each phase.

a p-value between acute and sub-acute phases.

b p-value between acute and convalescent phases.

*** p ≤ 0.001;

** p ≤ 0.01;

* p ≤ 0.05.

*SEM, standard error mean; CA, coronary artery; CA z-score MAX, largest coronary artery diameter between RCA and LAD z-scores; ns, not significant (p > 0.05)*.

### Increased Coronary Artery Z-Score Is Associated With Elevated Levels of PTX3 During Acute KD

**Figure 2:**
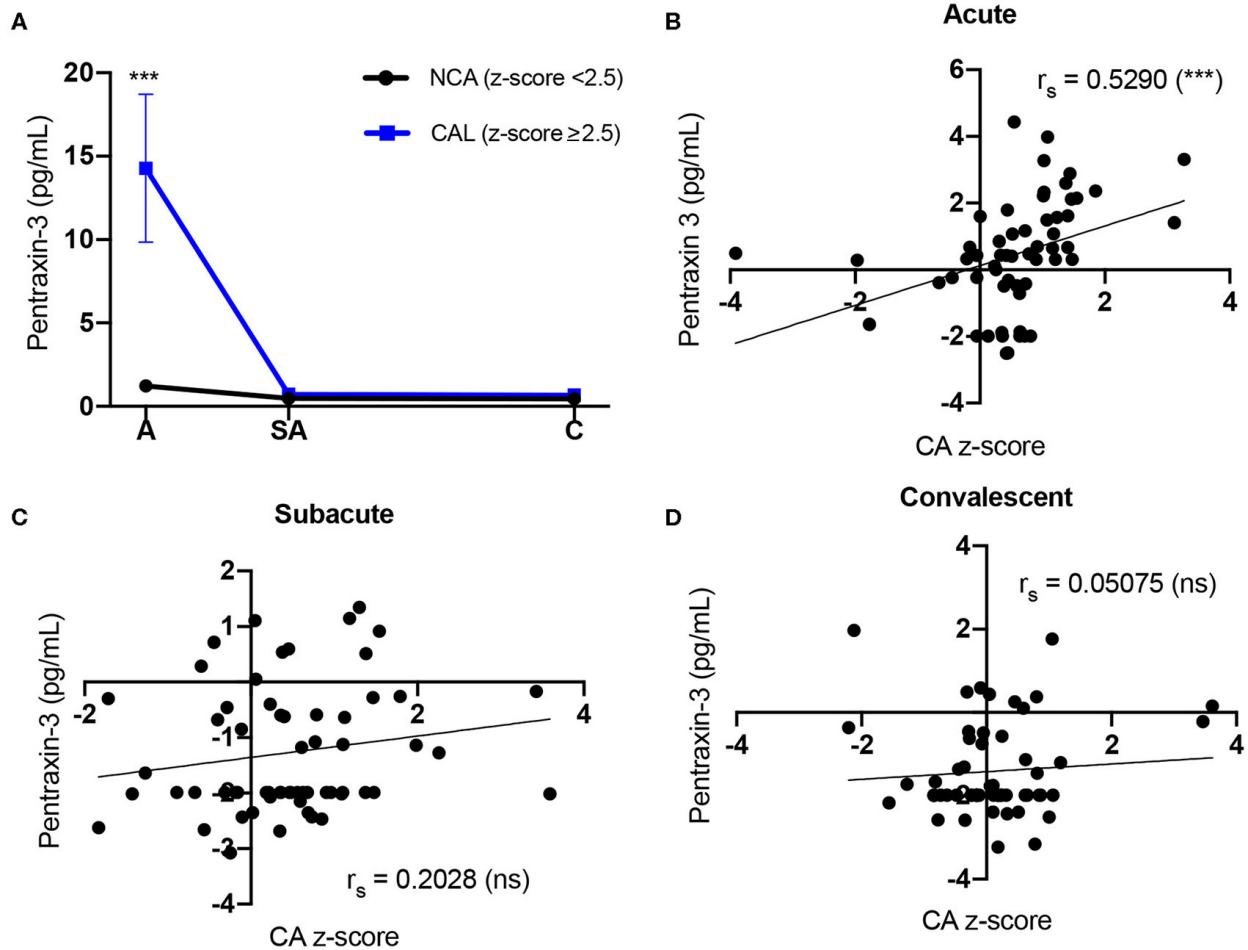
Comparison of Kawasaki disease (KD) patients' PTX-3 levels and coronary artery z-score. **(A)** Circulating PTX-3 levels in KD patients who develop coronary artery lesions (CAL) (z-score ≥ 2.5) and patients with normal coronary artery (NCA) (z-score <2.5). Repeated-measures ANOVA was used to evaluate group differences (i.e., CAL and NCA groups) of PTX-3 levels at the three phases of KD. Each graph represents the mean ± SEM for all the KD patients in the NCA (black line; *n* = 44, *A* = 39, SA = 40, *C* = 40) or CAL (blue line; *n* =26, A = 23, SA = 25, C = 24) groups. A, acute; SA, sub-acute; C, convalescent; SEM, standard error mean. **(B–D)** Spearman correlation between KD patients' circulating levels of PTX-3 and coronary artery z-score at the acute **(B)**, sub-acute **(C)**, and convalescent **(D)** phases of KD. The black line represents the best-fit linear regression between circulating PTX3 levels and coronary artery z-score. The variables were not normally distributed and thus were transformed by natural logarithm for analysis and plotting to reduce variance and satisfy model assumptions. *r*_s_, Spearman correlation analysis *r* value; A, acute (*n* = 62); SA, sub-acute (*n* = 65); C, convalescent (*n* = 64); CA, coronary artery; SEM, standard error mean; ns, not significant (*p* > 0.05). ***p ≤ 0.001.

We analyzed the PTX-3 levels in KD patients who developed CAL and compared them to KD patients with NCA. The KD patients who presented with a coronary artery z-score ≥2.5 in either LAD or RCA at any phase of the disease were assigned to the CAL group (*n* = 26), and all other KD patients were included in the NCA group (*n* = 44). During the acute phase, the PTX-3 levels (14.28 ± 4.44 pg/ml, 1.37–84.53) in the CAL group were significantly higher as compared to the NCA group (1.22 ± 0.21 pg/ml, 0.08–16.01) (Table 3). There was no significant difference in the PTX-3 levels between the CAL and the NCA groups at the sub-acute and the convalescent KD phases (Figure 2A, Table 3). The PTX-3 levels during acute KD in the NCA group remained significantly elevated as compared to the same group's convalescent phase (Table 3). When we compared the PTX-3 levels with the maximal coronary artery z-score at each phase of KD (largest coronary artery diameter of either LAD or RCA), we observed a significant correlation between the PTX-3 levels and the CA z-score during the acute KD phase (*r*_s_ = 0.529, 95% CI 0.304–0.698). However, this relationship was lost at the sub-acute and the convalescent KD phases (Figures 2B–D).

**Table 3:** PTX-3, coronary artery z-scores, and clinical assessment of inflammation throughout Kawasaki disease (KD) progression in patients with normal coronary arteries (NCA) and in patients who develop coronary artery lesions (CAL).

	**NCA (z-score** **<** **2.5) (*****n*** **=** **44)**											**CAL (z-score** **≥** **2.5) (*****n*** **=** **26)**											**NCA *vs*. CAL**
	**Acute (*****n*** **=** **44)**		***n***	**Sub-acute (*****n*** **=** **44)**		***n***	***P***^**a**^	**Convalescent (*****n*** **=** **41)**		***n***	***P***^**b**^	**Acute (*****n*** **=** **26)**		***n***	**Sub-acute (*****n*** **=** **26)**		***n***	***P***^**a**^	**Convalescent (*****n*** **=** **28)**		***n***	***P***^**b**^	***P***
PTX-3 (pg/ml)	1.22 ± 0.21	(0.08–16.01)	38	0.48 ± 0.11	(0.05–3.03)	40	^***^	0.46 ± 0.18	(0.04–7.18)	42	^***^	14.28 ± 4.44	(1.37–84.53)	21	0.73 ± 0.19	(0.10–3.84)	26	^***^	0.67 ± 0.23	(0.08–5.85)	25	^***^	^***^^c^, ns^d^^,^ ^e^
Right coronary artery z-score	0.66 ± 0.16	(−2.35–2.19)	44	0.47 ± 0.16	(−2.40–1.84)	44	ns	0.17 ± 0.14	(−1.92–2.23)	41	ns	3.27 ± 0.9	(−0.50–20.83)	26	3.39 ± 1.04	(−0.35–24.03)	26	ns	2.39 ± 1.19	(−1.25–25.77)	26	ns	*^c^^,^ ^d^,ns^e^
Left anterior descending coronary artery z-score	0.89 ± 0.17	(−3.00–2.50)	44	0.37 ± 0.17	(−3.00–2.44)	44	ns	0.26 ± 0.16	(−2.25–2.17)	41	ns	4.95 ± 1.18	(−0.60–26.24)	26	5.31 ± 1.69	(−0.09–36.30)	26	ns	3.84 ± 1.87	(−1.01–37.23)	26	^***^	^***^^c^^,^ ^d^, ns^e^
CA z-score MAX	1.18 ± 0.15	(−2.35–2.5)	44	0.89 ± 0.14	(−2.34–2.44)	44	ns	0.69 ± 0.11	(−1.00–2.23)	41	^***^	5.42 ± 1.13	(1.73–26.24)	26	5.82 ± 1.65	(0.85–36.30)	26	ns	4.21 ± 1.84	(0.48–37.23)	26	^***^	^***^^c^^,^ ^d^^,^ ^e^
Erythrocyte sedimentation rate (mm/h)	75.89 ± 4.26	(14.00 – 120.0)	44	84.35 ± 4.92	(6.00–120.0)	40	ns	13.2 ± 1.8	(1.00–59.00)	41	^***^	82.96 ± 5.63	(19.00–120.0)	24	86.81 ± 6.32	(20.00–120.0)	26	ns	16.36 ± 3.36	(1.00–84.00)	25	^***^	ns^c^^,^ ^d^^,^ ^e^
C-reactive protein (mg/L)	107.6 ± 12.8	(1.80–427.5)	44	7.26 ± 1.6	(0.00–42.60)	41	^***^	0.94 ± 0.31	(0.00–8.50)	36	^***^	114.7 ± 16.9	(7.40–310.7)	25	7.76 ± 1.94	(0.30–37.5)	25	^***^	1.45 ± 0.54	(0.00–11.40)	24	^***^	ns^c^^,^ ^d^^,^ ^e^
Hemoglobin (g/dl)	11.13 ± 0.16	(8.80–13.50)	44	10.91 ± 0.18	(8.60–13.30)	41	ns	12.14 ± 0.17	(8.90–14.80)	41	^***^	10.71 ± 0.23	(8.20–12.50)	26	10.41 ± 0.2	(8.30–12.00)	26	ns	12.19 ± 0.18	(10.50–13.60)	25	^***^	ns^c^^,^ ^d^^,^ ^e^
Hematocrit (g/dl)	33.15 ± 0.42	(27.10–38.40)	44	32.69 ± 0.47	(27.30–38.50)	41	ns	36.2 ± 0.45	(29.20–43.10)	41	^***^	31.64 ± 0.64	(25.60–36.30)	25	31.33 ± 0.59	(25.00–36.90)	26	ns	36.23 ± 0.44	(31.00–40.40)	25	^***^	ns^c^^,^ ^d^^,^ ^e^
Red blood cell (10^12^/L)	4.17 ± 0.06	(3.23–4.99)	44	4.08 ± 0.06	(3.14–4.90)	41	ns	4.52 ± 0.06	(3.69–5.35)	41	^***^	3.95 ± 0.09	(3.09–4.55)	25	3.85 ± 0.09	(2.89–4.49)	25	ns	4.6 ± 0.07	(3.87–5.14)	25	^***^	ns^c^^,^ ^d^^,^ ^e^
White blood cell (10^9^/L)	15.59 ± 0.97	(7.80–42.00)	44	12.25 ± 0.69	(4.20–25.60)	41	^**^	9.2 ± 0.41	(5.20–17.50)	41	^***^	13.92 ± 1.15	(3.10–28.10)	26	10.73 ± 0.77	(5.00–21.00)	26	ns	8.67 ± 0.45	(3.50–13.60)	25	^***^	ns^c^^,^ ^d^^,^ ^e^
Absolute lymphocyte count (10^9^/L)	3.39 ± 0.28	(0.39–7.17)	40	5.93 ± 0.35	(1.89–11.10)	40	^***^	5.41 ± 0.35	(2.14–11.55)	40	^***^	2.62 ± 0.4	(0.44–8.40)	23	5.32 ± 0.41	(2.32–8.69)	25	^***^	4.83 ± 0.4	(1.41–8.36)	24	^***^	ns^c^^,^ ^d^^,^ ^e^
Absolute neutrophil count (10^9^/L)	10.76 ± 1.02	(1.64–35.70)	40	5.13 ± 0.66	(0.84–17.66)	38	^***^	2.91 ± 0.28	(0.74–8.78)	40	^***^	12.43 ± 2.43	(1.09–63.20)	24	4.56 ± 0.66	(0.67–13.23)	25	^***^	3.17 ± 0.3	(0.78–7.07)	25	^***^	ns^c^^,^ ^d^^,^ ^e^
Absolute eosinophil count (10^9^/L)	0.47 ± 0.07	(0.03–1.38)	31	0.5 ± 0.07	(0.07–1.66)	35	ns	0.42 ± 0.07	(0.03–2.28)	37	ns	0.28 ± 0.06	(0.01–0.91)	18	0.35 ± 0.06	(0.01–1.21)	22	ns	0.31 ± 0.04	(0.04–0.92)	24	ns	ns^c^^,^ ^d^^,^ ^e^
Absolute basophil count (10/L)	0.07 ± 0.02	(0.02–0.41)	21	0.13 ± 0.03	(0.02–0.37)	13	ns	0.08 ± 0.01	(0.02–0.20)	24	ns	0.07 ± 0.02	(0.03–0.18)	8	0.07 ± 0.01	(0.03–0.10)	9	ns	0.08 ± 0.01	(0.03–0.19)	16	ns	ns^c^^,^ ^d^^,^ ^e^
Monocyte (10^9^/L)	0.78 ± 0.08	(0.12–2.45)	39	0.85 ± 0.08	(0.11–2.02)	39	ns	2.55 ± 0.5	(0.15–14.00)	40	ns	0.72 ± 0.12	(0.11–2.80)	22	0.58 ± 0.06	(0.10–1.09)	24	ns	2.15 ± 0.62	(0.07–13.00)	25	ns	ns^c^^,^ ^d^^,^ ^e^
PLT (10^9^/L)	380 ± 16.9	(150.0–650.0)	44	642.9 ± 23.4	(381.0–1,014)	41	^***^	380.9 ± 15.9	(228.0–743.0)	41	ns	388.1 ± 31.9	(68.00–699.0)	26	616.9 ± 45.5	(302.0–1,365)	26	^***^	379.8 ± 26.2	(207.0–737.0)	25	ns	ns^c^^,^ ^d^^,^ ^e^

Mean ± SEM (range) evaluated for the indicated variable of all the patient serum samples (n = 70) at the acute, sub-acute, and convalescent phases of the disease stratified by NCA and CAL. Repeated-measures ANOVA was used to compare the coronary artery z-scores and the clinical laboratory parameters between the three phases of KD. p-value comparing the effect of the variable at each phase.

a p-value between acute and sub-acute phases.

b p-value between acute and convalescent phases.

c p-value comparing the acute NCA and the acute coronary artery lesion groups.

d p-value comparing the sub-acute NCA and the sub-acute coronary artery lesion groups.

e p-value comparing the convalescent NCA and the convalescent coronary artery lesion groups.

*** p ≤ 0.001;

** p ≤ 0.01;

* p ≤ 0.05.

*SEM, standard error mean; CA, coronary artery; CA z-score MAX, largest coronary artery diameter between the right coronary artery and the left anterior descending coronary artery z-scores; ns, not significant (p > 0.05)*.

Subsequently, we compared the clinical laboratory parameters in CAL *vs*. NCA groups. None of the parameters evaluated revealed any significant differences between these two patient groups (Table 3, Supplementary Figure 2). The group stratification, however, did reveal some trends among the clinical laboratory parameters. The mean ESR, CRP, and ANC levels were higher in the CAL group as compared to the NCA group in the acute phase. Mean Hgb, Hct, RBC, and WBC levels were lower in the CAL group as compared to the NCA group in the acute phase (Table 3, Supplementary Figure 2).

### PTX-3 Levels Are Highly Correlated to Neutrophil Counts in KD

**Figure 3:**
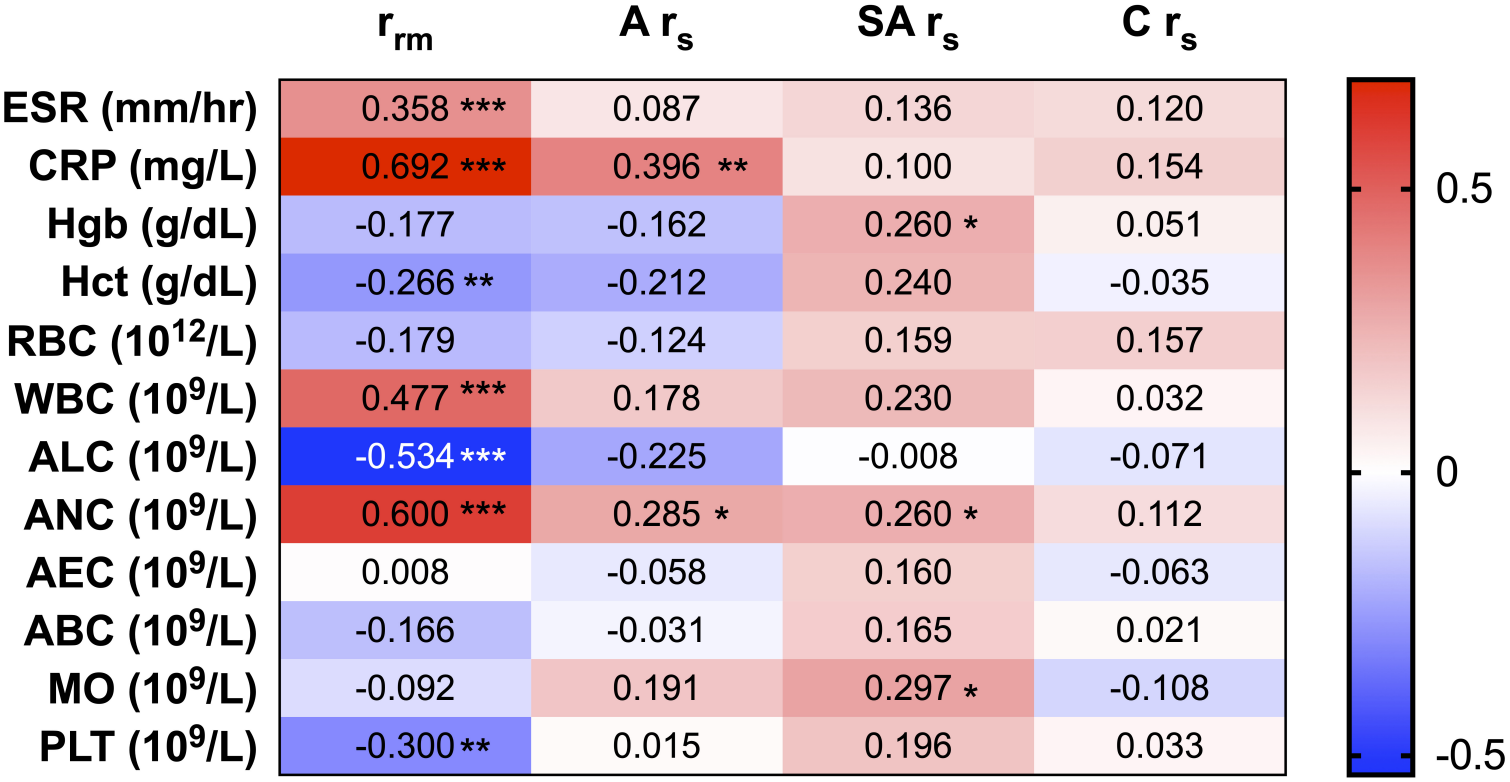
Correlations between PTX-3 and clinical measures of inflammation (i.e., C-reactive protein, erythrocyte sedimentation rate, and complete blood count). The number in each cell in the heat map indicates the repeated-measures correlation analysis (*r*_rm_) between PTX-3 and the clinical laboratory parameters of inflammation at all phases of Kawasaki disease (KD) and Spearman's correlation (*r*_s_) analysis between PTX-3 and the clinical laboratory parameters of inflammation at the acute (A *r*_s_), sub-acute (SA *r*_s_), and convalescent (C *r*_s_) phases of KD. The red cells show positive correlations and the blue cells show negative correlations. *p ≤ 0.05; **p ≤ 0.01; ***p ≤ 0.001.

To evaluate the role of PTX-3 in the inflammatory process of KD, we correlated PTX-3 to the clinical markers of inflammation. Longitudinal correlations between PTX-3 and clinical laboratory parameters by repeated-measures correlations (*r*_rm_) demonstrated a significant, strong positive correlation between PTX-3 and ESR, CRP, WBC, and ANC and significant, strong negative correlations with Hct, ALC, and PLT (Figure 3). At the individual phases of KD, we observed weaker Spearman correlations (*r*_s_) between PTX-3 and the clinical laboratory parameters (Figure 3). Moderately positive *r*_s_ correlations were observed between PTX-3 and CRP and ANC during the acute phase, weak positive *r*_s_ correlations were observed between PTX-3 and ANC, Hgb, and MO during the sub-acute phase, and no *r*_s_ correlations were observed in the convalescent phase (Figure 3).

### PTX-3 Levels and Clinical Laboratory Parameters of Inflammation Have Strong Positive Correlations in KD Patients With CAL

**Figure 4:**
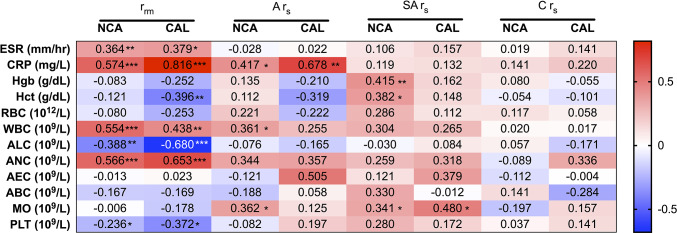
Correlations between PTX-3 and the clinical laboratory parameters of inflammation (i.e., C-reactive protein, erythrocyte sedimentation rate, and complete blood count) stratified by normal coronary artery (z-score <2.5) and coronary artery lesion (z-score ≥ 2.5) groups. The number in each cell in the heat map indicates the repeated-measures correlation (*r*_rm_) analysis between PTX-3 and the clinical assessment of inflammation at all phases of Kawasaki disease (KD) and Spearman's correlation (*r*_s_) analysis between PTX-3 and the clinical laboratory parameters of inflammation at the acute (A *r*_s_), sub-acute (SA *r*_s_), and convalescent (C *r*_s_) phases of KD. The red cells show positive correlations and the blue cells show negative correlations. *p ≤ 0.05; **p ≤ 0.01; ***p ≤ 0.001.

To further investigate the1 differences in PTX-3 levels between KD patients with and without CAL, we stratified the data between the CAL and the NCA patient groups and then conducted a correlation analysis between PTX-3 and the clinical laboratory parameters of inflammation (Figure 4). Stratifying the KD patients by coronary artery z-score to evaluate correlations between PTX-3 levels and thee clinical laboratory parameters of inflammation revealed differences in the repeated-measures correlation (*r*_rm_) and Spearman's correlation (*r*_s_) analyses between the two groups. Overall, the *r*_rm_ and *r*_s_ correlations were stronger in the CAL group. Similar to the un-stratified analysis, the *r*_rm_ analysis in both the NCA and the CAL groups showed a significant, strong positive correlation between PTX-3 and ESR, CRP, WBC, and ANC and significant, strong negative correlations with ALC and PLT (Figure 4). In the CAL group, there was a strong negative *r*_rm_ between PTX-3 and Hct, which was not observed in the NCA group (Figure 4). During the acute phase, *r*_s_ analysis between PTX-3 and CRP remained significantly positive in both groups. Analysis of the acute phase NCA group by *r*_s_ revealed weak positive relationships between PTX-3 and ALC and MO. Similarly, analysis of the sub-acute phase revealed positive *r*_s_ between PTX-3 and MO in both the NCA and the CAL groups. In the NCA group, sub-acute phase *r*_s_ analysis between PTX-3 and Hgb and Hct showed weak positive relationships. Furthermore, there were no *r*_s_ between PTX-3 and the clinical parameters of inflammation in the convalescent phase for the NCA and the CAL groups (Figure 4).

### Elevated Levels of PTX-3 and CAL Is Associated With IVIG Resistance

**Figure 5:**
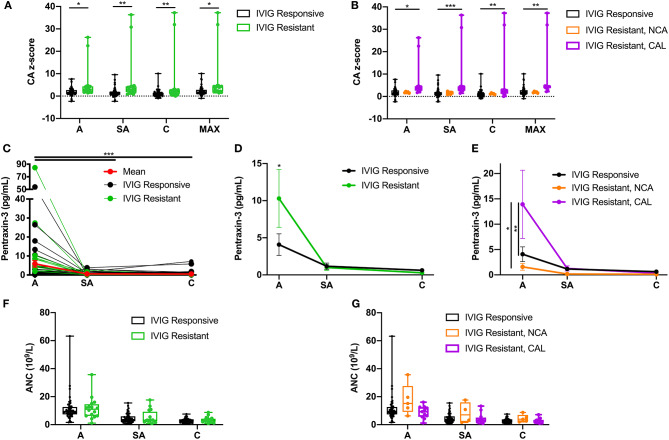
Effects of intravenous immunoglobulin (IVIG) response on coronary artery z-scores, circulating PTX-3 levels, and absolute neutrophil count (ANC). **(A)** Coronary artery z-score among Kawasaki disease (KD) patients responsive to IVIG treatment (black; *n* = 52) and resistant to IVIG treatment (green; *n* = 18) throughout the disease progression. **(B)** Coronary artery z-score among KD patients responsive to IVIG treatment (black; *n* =52) and resistant to IVIG treatment with normal coronary artery (NCA) (orange; *n* = 5) and IVIG resistant with coronary artery lesion (CAL) (purple; *n* =13) throughout the disease progression. **(C)** Circulating PTX-3 levels in single patients throughout the KD progression: A, *n* = 62; SA, *n* = 65; and C, *n* = 64. The black lines track KD patients who were responsive to IVIG treatment (*n* = 52; A = 40, SA = 47, and C = 48), the green lines track KD patients who were resistant to IVIG treatment (*n* = 18, A = 16, SA = 18, and C = 16), and the red line track the mean ± SEM of all the patient serum samples (*n* = 70) evaluated at each phase of the disease. **(D)** Circulating levels of PTX-3 in KD patients responsive (black, *n* = 52) or resistant (green, *n* = 18) to IVIG treatment throughout the disease progression. **(E)** Circulating levels of PTX-3 in KD patients responsive to IVIG treatment (*n* = 52; A = 40, SA = 47, and C = 48), resistant to IVIG treatment with NCA (*n* = 5 for all three KD phases), and resistant to IVIG treatment with CAL (*n* = 13; A = 11, SA = 13, and C = 11). **(F)** ANC among KD patients responsive to IVIG treatment (black; *n* = 52, A = 48, SA = 48, and C = 48) and resistant to IVIG treatment (green; *n* = 18, A = 16, SA = 16, and C = 15) throughout the disease progression. **(G)** ANC among KD patients responsive to IVIG treatment (black; *n* = 52, A = 48, SA = 48, and C = 48), resistant to IVIG treatment with NCA (orange; *n* = 5, A = 5, SA = 4, and C = 5), and IVIG resistant with CAL (purple; *n* = 13, A = 11, SA = 12, and C = 12) throughout the disease progression. The box plots mean, 25, 75%, minimum, maximum, and individual points of all patients' (*n* = 70) maximal coronary artery z-score among the right coronary artery and left anterior descending coronary artery at the acute, sub-acute, and convalescent phases of KD, stratified by IVIG response (i.e., responsive or resistant) and coronary artery z-score (i.e., NCA or CAL). Repeated-measures ANOVA was used to compare the IVIG treatment response groups with NCA or CAL, with coronary artery z-scores, circulating PTX-3 levels, and ANC at the three phases of KD and the maximal coronary artery z-score throughout KD progression. C, convalescent; SEM, standard error mean; CA, coronary artery; MAX, largest coronary artery diameter between RCA and LAD z-scores; A, acute; SA, sub-acute. *p*-value comparing the effect of the IVIG treatment response groups at each phase. ***p ≤ 0.001; **p ≤ 0.01; *p ≤ 0.05.

IVIG resistance is a well-known risk factor for CAL. Our study population included 18 (26%) IVIG-resistant patients (Table 1), of whom 13 (72%) also exhibited CAL. Fisher's exact test confirmed that IVIG resistance was a risk factor for CAL (OR = 7.8; 95% CI 2.38–23.54, *p* < 0.001) in this study. Comparisons of the coronary artery z-scores between IVIG-responsive and IVIG-resistant patients demonstrated significantly larger coronary artery z-scores throughout KD progression among IVIG-resistant patients as compared to IVIG-responsive patients (Figure 5A, Table 4). These differences were more pronounced by further stratification of IVIG resistance among KD patients with NCA and CAL. The coronary artery z-scores among IVIG-resistant KD patients with CAL were significantly larger than those of KD patients responsive to IVIG treatment at all phases of the disease (Figure 5B, Table 4). There were no statistically significant differences in the coronary artery z-score among IVIG-resistant groups with NCA or CAL.

**Table 4:** Coronary artery z-scores, PTX-3 levels, and absolute neutrophil counts (ANC) throughout Kawasaki disease (KD) progression.

			**Intravenous immunoglobulin (IVIG) susceptible (*****n*** **=** **52)**		***n***	**IVIG resistant (*****n*** **=** **18)**		***n***	***P*^**a**^**		**IVIG resistant, NCA (*****n*** **=** **5)**		***n***	**IVIG resistant CAL (*****n*** **=** **13)**		***n***	***P*^**b**^**	***P*^**c**^**
CA z-score	Acute	*n* = 70	1.87 ± 0.25	(−2.35–7.63)	52	5.32 ± 1.66	(1.42–26.24)	18	^*^	*n* = 70	1.81 ± 0.15	(1.42–2.25)	5	6.66 ± 2.2	(1.73–26.24)	13	^*^	ns
	Sub-acute	*n* = 70	1.53 ± 0.27	(−2.34–9.57)	52	6.17 ± 2.37	(0.83–36.30)	18	^**^	*n* = 70	1.55 ± 0.3	(0.83–2.34)	5	7.94 ± 3.18	(1.43–36.30)	13	^***^	ns
	Convalescent	*n* = 70	1.11 ± 0.23	(−1.00–10.09)	52	5.38 ± 2.52	(0.00–37.23)	18	^**^	*n* = 70	1.18 ± 0.15	(0.71–1.66)	5	7 ± 3.42	(0.00–37.23)	13	^**^	ns
	CA z-score MAX	*n* = 70	2.21 ± 0.27	(−1.00–10.09)	52	6.76 ± 2.41	(1.42–37.23)	18	^*^	*n* = 70	1.87 ± 0.16	(1.42–2.34)	5	8.64 ± 3.21	(2.16–37.23)	13	^**^	ns
PTX-3 (pg/ml)	Acute	*n* = 62	4.08 ± 1.46	(0.08–53.92)	40	10.29 ± 4.9	(0.14–84.53)	16	^*^	*n* = 62	1.59 ± 0.66	(0.14–3.24)	5	13.92 ± 6.73	(1.37–84.53)	11	^**^	^*^
	Sub-acute	*n* = 65	1.17 ± 0.43	(0.05–19.10)	47	0.99 ± 0.39	(0.09–7.23)	18	ns	*n* = 65	0.18 ± 0.06	(0.09–0.43)	5	1.28 ± 0.51	(0.13–7.23)	13	ns	ns
	Convalescent	*n* = 64	0.62 ± 0.19	(0.04–7.18)	48	0.28 ± 0.07	(0.08–1.17)	16	ns	*n* = 64	0.12 ± 0.02	(0.08–0.17)	5	0.35 ± 0.1	(0.14–1.17)	11	ns	ns
Absolute neutrophil count (10^9^/L)	Acute	*n* = 64	11.31 ± 1.32	(1.64–63.2)	48	11.63 ± 2	(1.09–35.7)	16	ns	*n* = 64	17.76 ± 4.98	(6.32–35.70)	5	8.84 ± 1.32	(1.09–16.04)	11	ns	ns
	Sub-acute	*n* = 64	4.75 ± 0.49	(0.84–15.53)	48	5.34 ± 1.23	(0.67–17.66)	16	ns	*n* = 64	8.32 ± 3.71	(1.56–17.66)	4	4.35 ± 1.07	(0.67–13.23)	12	ns	ns
	Convalescent	*n* = 64	2.87 ± 0.22	(0.74–7.55)	48	3.39 ± 0.5	(0.78–8.78)	15	ns	*n* = 64	4.46 ± 1.21	(1.96–8.78)	5	2.94 ± 0.48	(0.78–7.07)	12	ns	ns

Mean ± SEM (range) evaluated for the indicated variable of all the patient serum samples (n = 70) evaluated at the acute, sub-acute, and convalescent phases of the disease in IVIG responsive, IVIG resistant, IVIG resistant with normal coronary artery (NCA), and IVIG resistant with coronary artery lesion (CAL). Repeated-measures ANOVA was used to compare the coronary artery z-scores, circulating PTX-3 levels, and absolute neutrophil counts among IVIG response groups between the three phases of KD. p-value comparing the effect of the IVIG response groups for coronary artery z-score, PTX-3 levels, and ANC.

a p-value between IVIG-responsive and IVIG-resistant KD patients.

b p-value between IVIG-responsive and IVIG-resistant with CAL KD patients.

c p-value between IVIG-resistant with NCA and IVIG-resistant with CAL KD patients.

*** p ≤ 0.001;

** p ≤ 0.01;

* p ≤ 0.05.

*SEM, standard error mean; CA, coronary artery; CA z-score MAX, largest coronary artery diameter the right coronary artery and the left anterior descending coronary artery z-scores; ns, not significant (p > 0.05)*.

Next, we evaluated the IVIG response group effects on the circulating levels of PTX-3 (Figure 5C, Table 4). The PTX-3 levels were significantly elevated during acute KD in IVIG-resistant patients as compared to those IVIG-responsive patients (Figure 5D, Table 4). There were no statistically significant differences in the PTX-3 levels among IVIG-responsive and IVIG-resistant KD patients at the sub-acute and the convalescent phases of the disease. The PTX-3 levels were further stratified in the NCA and the CAL groups among the IVIG-resistant groups. The PTX-3 levels during the acute phase were significantly higher in KD patients who were IVIG resistant with CAL as compared to those in KD patients who were IVIG resistant with NCA and in KD patients responsive to IVIG treatment. However, there were no significant differences between KD patients resistant to IVIG with NCA and KD patients responsive to IVIG treatment (*p* = 0.99) (Figure 5E, Table 4). An investigation of the effect of IVIG response in NCA and CAL patients on ANC did not reveal any statistically significant differences (Figures 5F,G, Table 4).

## Discussion

PTX-3 is produced in response to proinflammatory signals and microbial stimulation and by a wide variety of immune and endothelial cells with roles in the regulation of inflammation and complement activation as well as in vascular inflammation and endothelial cell dysfunction (23, 32). Elevated levels of PTX-3 have been found in septic shock (33, 34), chronic kidney disease (35), stroke (36), and a variety of cardiovascular diseases (25, 37, 38). In younger populations, elevated levels of PTX-3 have been associated with neonatal sepsis (39), severe pediatric microbial infections (40, 41), and autoimmune diseases, such as childhood-onset systemic lupus erythematosus (42), juvenile idiopathic arthritis (43), and asthma (44). In this study, we found significantly elevated circulating levels of PTX-3 in a cohort of patients with acute KD as compared to the PTX-3 levels in the same patients upon resolution of the disease, i.e., convalescent phase. Furthermore, we observed a significant difference in PTX-3 levels in patients with CAL when compared to patients without CAL during acute KD.

### PTX-3 in KD Is Correlated to Clinical Assessment of Inflammation

Consistent with previous studies of clinical laboratory data in KD progression (45–48), in our study, the levels of ESR, CRP, WBC, and ANC peaked in the acute phase, the platelet levels were highest in the sub-acute phase, and RBC, Hct, Hgb, and leukocyte levels were highest in the convalescent phase. Correlations between individual KD patient's clinical laboratory data and the PTX-3 levels throughout KD progression showed PTX-3 levels with positive correlations to ESR, CRP, WBC, and ANC levels and negative correlations with Hct, ALC, and PLT. During the acute phase, the PTX-3 levels were positively correlated to CRP and ANC. At the sub-acute phase, the PTX-3 levels were positively correlated to Hgb, ANC, and MO. No clinical laboratory parameters were correlated to the PTX-3 levels in the convalescent phase, consistent with PTX-3 as an inflammatory modulator during acute KD pathogenesis. The ESR and the CRP levels are established inflammatory parameters (30). Strong repeated-measures correlation analysis between PTX-3 and these clinical inflammatory markers suggests a role for PTX-3 in monitoring KD disease progression and further supports the diagnostic and the prognostic potential of PTX-3 for KD diagnosis and risk scoring.

### Neutrophils as a Source of PTX-3 During KD Progression

The positive repeated-measures correlation and Spearman's correlation in the acute phase between WBC, specifically neutrophils and PTX-3, suggest neutrophils as a possible source for PTX-3. Neutrophils have been shown to release PTX-3 when activated in cardiovascular disease and sepsis (33, 34, 39). Pathology studies of heart tissues from autopsies of children with KD has led to a model of KD vasculopathy that begins with an initial neutrophilic infiltration of the coronary artery, followed by infiltration of monocytes and macrophages, implicating these immune cells as key players in KD-associated CAL and vascular dysfunction (7, 8, 49, 50). Macrophages, dendritic cells, and endothelial cells express PTX-3 in response to lipopolysaccharides, interleukin (IL)-1 and −1β, and tumor necrosis factor (TNF)-α (51, 52). Similarly, PTX-3 is released from neutrophil granules in response to tissue damage (53). Interestingly, TNF-α (54) and IL-1β (55, 56) are recognized biomarkers in acute KD. Inflammatory biomarkers, immunological markers, and proteomic biomarkers have been demonstrated to be elevated in the blood of acute KD patients (22). In particular, elevated levels of the neutrophil-derived S100A12 protein, a pro-inflammatory ligand for the PRR receptor for advanced glycation end products (RAGE), were negatively regulated by soluble RAGE (57–60). Our data suggest that PTX-3 is among the neutrophil products that make up the inflammatory milieu in the coronary arteries of KD patients. Furthermore, previous studies of KD pathogenesis and PTX-3 expression suggest that neutrophils, in response to induction by IL-1β and TNF-α, induce PTX-3 production in the coronary artery during acute KD. Therefore, we propose that PTX-3 may play an important role in KD pathogenesis, particularly in vascular dysfunction leading to CAL.

### PTX-3 in KD-Associated CAL

It has been suggested that PTX-3 plays dual roles, both protective and harmful, in the development and the progression of a cardiovascular disease (25, 28, 61). Previous studies have implicated a variety of stimuli to induce PTX-3 expression/release in immune and vascular endothelial cells (49, 50). Our analysis of the potential group effects in clinical laboratory parameters did not reveal any significant differences in the clinical laboratory parameters of KD patients with and without CAL. PTX-3 may have a different site-specific function, which could explain the pro-inflammatory and the anti-inflammatory roles of PTX-3 in cardiovascular diseases (62). PTX-3 decreases nitrogen oxide synthesis in endothelial cells, reducing cell proliferation and function and thus promoting endothelial dysfunction (63, 64). Similarly, PTX-3 inhibits angiogenesis through inhibition of fibroblast growth factor-2 that alters several functions including inflammation, tissue repair, and growth (65, 66). Furthermore, PTX-3 interacts with P-selectin, promoting lymphocyte recruitment, vascular inflammation, and endothelial dysfunction that can result in morphological alterations (28). Taken together, these observations and our data suggest the value of evaluating the PTX-3 levels in KD patients as other clinical assessments of inflammation fail to distinguish KD patients with CAL from those without CAL.

There have been several studies investigating PTX-3 in the context of coronary artery disease, with conflicting results. In nine studies, published between 2004 and 2017, among adult study populations conducted in Asia, Europe, and North America, there seems to be a consensus that patients with coronary disease and higher circulating PTX-3 levels had an increased risk of all-cause mortality, cardiac death, and cardiac events (26). In our study, we observed significantly higher PTX-3 levels in patients with CAL as compared to patients without CAL during the acute, but not in the sub-acute nor the convalescent, phase of KD. Comparisons between individual KD patient's coronary artery z-score to their PTX-3 levels revealed strong positive correlations between the clinical laboratory parameters in the acute phase, which were lost in the sub-acute and the convalescent phases of KD.

Stratification of KD patients by coronary artery z-scores in the CAL and the NCA groups revealed a stronger repeated-measures correlation between PTX-3 and the clinical assessments of inflammation in the CAL group. Previous studies have observed elevated sub-acute and convalescent WBC and ESR levels (46) and higher sub-acute PLT levels (67) associated with KD patients with CAL. Furthermore, elevated levels of PTX-3 in acute KD remained significant among both NCA and CAL groups. While the group stratification implicates the role of PTX-3 in the development of CAL, the protein is not exclusive to this process; rather, it is integral in the overall progression of KD. Thus, research in adult human coronary artery disease (26) in conjunction with our data implicates PTX-3 as a modulator of vascular dysfunction and remodeling and could have a specific role in KD-associated CAL. Therefore, we propose PTX-3 as a sensitive marker for coronary artery dilation in KD.

### PTX-3 in KD-Associated IVIG Resistance

IVIG resistance is a risk factor for the development of CAL in KD (30). Fever status is an indicator of systemic inflammation. Patients resistant to IVIG treatment experience prolonged systemic inflammation, which includes inflammation of the coronary arteries, leading to an increased risk for CAL (68, 69). Among our patient population, the KD patients resistant to IVIG treatment were at a higher risk for CAL as compared to the KD patients who were responsive to IVIG treatment.

Researchers in Japan who evaluated the transcriptional regulation of infliximab therapy in IVIG-resistant KD patients identified high levels of PTX-3 transcript levels in IVIG-resistant KD patients (70). Therefore, we stratified our data on the circulating levels of PTX-3 among IVIG-responsive and IVIG-resistant KD patients and confirmed that the PTX-3 levels were higher in the IVIG-resistant patients as compared to those in the IVIG-responsive patients. These differences in the circulating PTX-3 levels were more dramatic when further stratifying the IVIG resistance group by coronary artery z-score in the NCA and the CAL groups. The circulating PTX-3 levels in the IVIG-resistant patients with CAL were significantly elevated when compared to the IVIG-resistant patients with NCA. Furthermore, the PTX-3 levels in the IVIG-responsive group were similar to those in the IVIG-resistant patients with NCA. Collectively, these data suggest that the elevated levels of PTX-3 are indicative of enlarged coronary arteries rather than IVIG resistance.

Studies of clinical laboratory markers have described elevated CRP, liver enzyme level, WBC, and neutrophil counts to be associated with KD patients resistant to IVIG treatment (68). Our data implicate the role of PTX-3 in producing the heightened inflammatory environment in IVIG-resistant KD patients with CAL. Previous studies have suggested elevated neutrophil counts in IVIG resistance (17, 18, 71). However, we and others (72, 73) did not observe elevated neutrophil counts in either IVIG-resistant patients or IVIG-resistant patients with CAL as compared to those in IVIG-responsive patients. Perhaps our small and heterogeneous patient population may account for these differences in the neutrophil counts. Furthermore, most data on the positive correlation of IVIG resistance and neutrophil counts are from homogenous populations (16–18, 71).

### Patient Population

In this study, we demonstrate the potential role of PTX-3 in KD pathogenesis and particularly in coronary dilation that is the most significant outcome of the disease. A strength of these findings is that they have been obtained in a population of mixed ethnicity. Ethnicity is a well-known risk factor for KD and KD-associated CAL. Much KD research has been conducted in ethnically homogenous study populations (i.e., Asian or White). Our study's geographic setting in Hawai‘i has resulted in a heterogeneous study population, including a majority of mixed-race patients. Perhaps this allows the associations that we detect to have more robust disease implication rather than geographic or ethnic specificity.

### Limitations and Future Directions

The limitations of this study include limited sample size, particularly within the CAL group, and some incomplete sampling of patients at a few time points. Convalescent phase samples were used as individual patient controls; however, the diagnosis of KD requires distinguishing KD patients from other febrile children admitted to the emergency room who present with similar symptoms (i.e., rash, inflammation of the mucous membranes, edema, conjunctivitis, and lymphadenopathy). Thus, additional studies investigating the specific role of PTX-3 in KD patients as compared to febrile and afebrile controls are warranted to evaluate the diagnostic potential of PTX-3. During normal physiological conditions, PTX-3 has been demonstrated as a non-specific systemic inflammatory protein known to have very low to undetectable levels in the circulation. The serum PTX-3 levels dramatically increase within 6–8 h of infection and inflammation (24). Thus, the non-specific role of PTX-3 in infection and inflammation might preclude the protein's utility as a general biomarker to distinguish KD patients from other febrile children. However, our results demonstrate a significant acute elevation of PTX-3 level among KD patients with CAL as compared to those with NCA. This data further suggests a specific role for PTX-3 in KD immunopathogenesis, particularly in CAL development. Future studies should focus on investigating the underlying mechanisms by which PTX-3 and other inflammatory proteins are involved in KD pathogenesis and CAL development.

## Conclusions

Currently, KD diagnosis relies on the identification of specific symptoms by an experienced clinician. Without prompt diagnosis and administration of treatment, KD patients are at an increased risk for severe complications such as CAL and aneurysm formation, which may result in permanent coronary vasculopathy and life-long risk for cardiovascular diseases (9). Therefore, there is a pressing need for better, less subjective diagnostic and prognostic methods for the identification of children with KD and those children who are at a greatest risk for cardiovascular complications associated with the disease. The current findings demonstrate the potential role of PTX-3 in the inflammatory process of acute KD and, in particular, as a contributor to CAL.

## Data Availability Statement

The raw data supporting the conclusions of this article will be made available by the authors, without undue reservation.

## Ethics Statement

This study was approved by the Kapi'olani Medical Center for Women and Children (KMCWC) Institutional Research and Ethics Committee (Western Consortium IRB Study No. 1140512). Informed consent was obtained from the parents or guardians of all patients prior to enrollment and specimen collection.

## Author Contributions

AB, MM, VN, and RS conceptualized and designed the study and interpreted the data. LC and VN conceptualized and designed the PTX-3 study. LC acquired the data, conducted an initial statistical analysis of the data, interpreted the data, and drafted the initial manuscript. VN supervised the data collection, interpreted the data, and drafted the initial manuscript. EL conducted a formal statistical analysis of the data. All authors reviewed and edited the final manuscript and have read and agreed to the published version of the manuscript.

## Conflict of Interest

The authors declare that the research was conducted in the absence of any commercial or financial relationships that could be construed as a potential conflict of interest.
