# Supplementary material for: Elevated Levels of Pentraxin 3 Correlate With Neutrophilia and Coronary Artery Dilation During Acute Kawasaki Disease
Source: Front Pediatr. 2020 Jun 25;8:295. doi: 10.3389/fped.2020.00295 (PMC7330095; doi:10.3389/fped.2020.00295)
Supplement: Supplementary file 1 [file Data_Sheet_1.docx]

Supplementary Material

## Supplementary Figures

**Supplementary Figure 1.** Coronary artery z-scores of (**A**) RCA, (**B**) LAD, (**C**) CA MAX, and (**D-O**) clinical laboratory parameters throughout KD progression. Box plots mean, 25%, 75%, minimum, maximum, and individual points of all the patient serum samples (n=70) evaluated at acute, sub-acute, and convalescent phases of the disease. Repeated measure ANOVA was used to compare coronary artery z-scores and clinical laboratory parameters between the three phases of KD. p-value comparing effect of the variable at each phase; *** p ≤ 0.001, ** p ≤ 0.01, * p ≤ 0.05, ns = not significant p > 0.05. A = acute; SA = sub-acute; C = convalescent; CA = coronary artery; CA z-score MAX = largest coronary artery diameter between RCA and LAD z-scores.

**Supplementary Figure 2.** CAL verses NCA group differences of coronary artery z-scores, (A) RCA, (B) LAD, (C) CA MAX, and (D-O) clinical laboratory parameters throughout KD progression. Box plots mean, 25%, 75%, minimum, maximum, and individual points of all the patient serum samples (n=70) evaluated at acute, sub-acute, and convalescent phases of the disease stratified by NCA (black; n=44) and CAL (blue; n=26) groups. Repeated measures ANOVA was used to compare coronary artery z-scores and clinical laboratory parameters between the three phases of KD. p-value comparing effect of the variable at each phase; *** p ≤ 0.001, ** p ≤ 0.01, * p ≤ 0.05, ns = not significant p > 0.05. A = acute; SA = sub-acute; C = convalescent; CA = coronary artery; CA z-score MAX = largest coronary artery diameter between RCA and LAD z-scores.
